# Clinical Validation of Commercial AI Software for the Detection of Incidental Vertebral Compression Fractures in CT Scans of the Chest and Abdomen

**DOI:** 10.3390/diagnostics15121530

**Published:** 2025-06-16

**Authors:** Vinu Mathew, Dawn Pearce, Noah Kates Rose, Sidharth Saini, Earl Bogoch

**Affiliations:** 1 Department of Medical Imaging, University of Toronto, Toronto, ON M5T 1W7, Canada; noah.katesrose@mail.utoronto.ca; 2 Department of Medical Imaging, Musculoskeletal Division, St. Michael’s Hospital, Unity Health Toronto, Toronto, ON M5B 1W8, Canada; dawn.pearce@unityhealth.to (D.P.); sid.saini1@gmail.com (S.S.); 3 Department of Surgery, University of Toronto, Toronto, ON M5S 1A1, Canada; earl.bogoch@unityhealth.to; 4 Li Ka Shing Knowledge Institute of St. Michael’s Hospital, Unity Health Toronto, Toronto, ON M5B 1W8, Canada

**Keywords:** vertebral compression fractures, artificial intelligence, osteoporosis, spine imaging

## Abstract

**Background/Objectives:** The objective of this study was to clinically validate the performance of the Nanox.AI HealthOST software in detecting incidental vertebral compression fractures (VCFs) on outpatient chest and abdomen CT scans using sensitivity, specificity, positive predictive value (PPV), and negative predictive value (NPV). A secondary aim was to assess the rate of missed VCFs using initial radiologist reports. **Methods:** A retrospective analysis was performed on 590 outpatient CT scans. HealthOST, an artificial intelligence solution from Nanox.AI that allows for automated spine analysis using CT images was evaluated against a consensus ground truth established by two radiologists, including a senior musculoskeletal radiologist. Two vertebral body height reduction thresholds were tested: mild (>20%) and moderate (>25%). Original radiologist reports were reviewed to identify missed VCFs. **Results:** At the 20% threshold, the AI achieved a sensitivity of 92.0%, a specificity of 52.7%, a PPV of 16.5%, and an NPV of 98.5%. At the 25% threshold, sensitivity decreased to 78.0%, while specificity improved to 94.2%, with a PPV of 51.1% and an NPV of 98.2%. The AI identified 88% and 92% of fractures missed by radiologists at the 20% and 25% thresholds, respectively. **Conclusions:** The Nanox HealthOST AI solution demonstrates potential as an effective screening tool, with threshold selection adaptable to clinical needs with a secondary review by a radiologist that is advisable to ensure diagnostic accuracy. The study further indicates that radiologists often overlook VCFs in reporting non-indicated cases and that AI has a role in enhancing the detection and reporting of vertebral compression fractures in routine clinical practice.

## 1. Introduction

With the rising prevalence of osteoporosis in Canada and globally, vertebral compression fractures represent a growing public health concern for fractures associated with the disease [1,2,3,4,5]. Osteoporosis-related fractures can greatly impact a person’s overall well-being and quality of life [6,7,8,9]. Osteoporosis-related fractures substantially contribute to the health care burden through high rates of hospitalization, rehabilitation needs, and the increased likelihood of long-term disability and dependence on extended care services [10,11,12,13]. Patients with osteoporotic fractures have demonstrated up to a 5-fold increased fracture risk within 2 years post primary fracture [14,15,16,17].

Vertebral compression fractures (VCFs) are among the most common osteoporotic fractures noted [18,19,20]. The 5-year survival rate post vertebral body fractures can be as low as 28%, potentially due to deteriorating symptoms and functional status [21,22,23]. Up to two-thirds of VCFs are incidental findings initially identified through imaging, but the majority of the VCFs on Computed Tomography (CT) scans remain incompletely reported or missed [20,24,25]. This is due to the fact that these fractures are often asymptomatic [25,26,27]. Early detection and intervention can provide significant benefits to patients by preventing future fractures, alleviating symptoms, and reducing morbidity and mortality [18,28]. Even after identifying the fractures, clinical management is often inadequate. When compared to acute coronary events, 90% of patients receive secondary preventive care while only 10–20% of individuals with osteoporotic fragility fractures are prescribed appropriate medications to reduce the risk of future fractures [5,14,29]. To address the gaps in VCF detection and management, multiple approaches have been proposed. Nanox HealthOST V1.1 software is an artificial intelligence (AI) software approved by Health Canada and the FDA that has demonstrated promise in opportunistically detecting VCFs on CT scans performed for unrelated diagnostic purposes.

The primary aim of this study is to validate the performance of Nanox.AI’s HealthOST software in detecting incidental vertebral compression fractures (VCFs) on chest and abdomen CT scans and evaluating the specificity, sensitivity, positive predictive value, and negative predictive value of the software in its detection. A secondary objective is to determine the prevalence of missed VCFs in outpatient CT scans at our institution. Recognizing previously undiagnosed vertebral fractures is clinically important as it signals a heightened risk for future fragility fractures. Early identification may lead to timely prophylactic treatment with bone-strengthening medications, as recommended by clinical guidelines, which will potentially reduce future fracture risks and associated morbidity and mortality. This study is significant in that it verifies the effectiveness of AI technology that has already been approved and commercialized, emphasizing its practical application as a reliable diagnostic aid in routine clinical settings.

## 2. Materials and Methods

This retrospective study involves the selection of 675 outpatient cases from St. Michael’s hospital, spanning from February 2019 to March 2020. The de-identified CT data was analyzed using HealthOST, an AI solution by Nanox.AI designed for automatic image analysis of the spine. This provides a tool for clinicians for the evaluation of indicators of osteoporosis and for detecting VCFs. The Nanox software’s results were evaluated using two different detection thresholds: mild (>20% vertebral height reduction) and moderate (>25% vertebral height reduction). These thresholds were used to compare the software’s findings with the radiologists’ assessments.

Following the initial AI analysis, two experienced radiologists reviewed all scans together and reached a consensus, establishing a single ground truth. The first reviewer was a senior musculoskeletal (MSK) radiologist, while the second was a fellowship-trained emergency radiologist with extensive experience in diagnosing vertebral fractures in trauma settings. Discrepancies were resolved through consensus discussions between both radiologists. In particularly complex cases, additional input was sought from a highly regarded colleague specializing in orthopedic surgery and metabolic bone disease to refine fracture classification and ensure diagnostic accuracy. After establishing the ground truth, we compared it to the AI results and reviewed the initial radiology report for any missed fracture detections.

The radiologists employed the Genant semiquantitative (GSQ) grading scheme, supplemented by quantitative morphometry (QM) for fractures where the actual height loss was measured. The actual measurement was taken in the anterior, mid, or posterior segment of the vertebral body and compared to the ratio of the corresponding segment of the closest normal vertebral body above or below. The severity of the fractures was graded using the GSQ grading scale as follows: grade 0, less than 20% height loss; grade 1, 20–25% height loss; grade 2, 26–40% height loss; and grade 3, more than 40% height loss [30]. Fractures were distinguished from non-fracture deformities by assessing endplate disruptions and vertebral body cortical buckling. The modified morphological algorithm-based qualitative (mABQ) method was not formally adopted as the current machine learning system Nanox.AI cannot reliably detect these morphological criteria.

The inclusion criteria for this study encompass outpatients who underwent chest and/or abdomen/pelvis CT scans at St. Michael’s hospital from February 2019 to March 2020. Participants were enrolled consecutively based on the chronological order of their CT scan dates and times to minimize selection bias. Only patients over the age of 50 were considered. The selection of the cutoff date, 1 March 2020, was intentional to exclude any potential confounding effects of the COVID-19 pandemic. This study was limited to outpatient CT scans to specifically assess our secondary objective, which was to evaluate incidental vertebral fractures that typically go unnoticed in the outpatient setting. In contrast, inpatient and emergency scans often involve acute trauma cases with higher clinical suspicion of fractures and more deliberate reporting. Additionally, we selected individuals aged ≥50 years to enrich the population with patients at greater risk of osteoporosis and vertebral compression fractures, thereby aligning with the intended clinical use case for opportunistic screening.

The exclusion criteria comprised patients younger than 50 years, those with spinal hardware fixation, and cases where the CT scan report lacked an available clinical indication or had indications related to assessments for vertebral body fractures. CT scans composed of excessive artifacts such as beam hardening and motion artifacts were also excluded. CT scans that did not have an adequate number of vertebral bodies to visualize the thoracic or lumbar spine were also excluded. Patients with preexisting medical conditions were not excluded.

**Figure 1:**
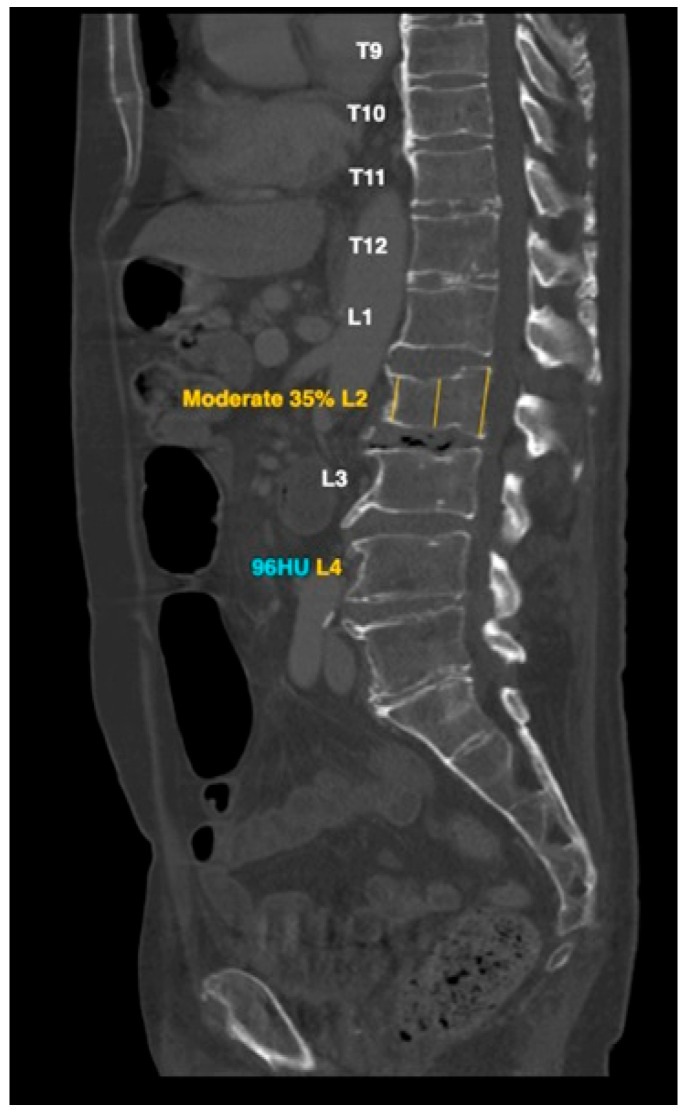
AI-based L2 vertebral compression fracture calculation and attenuation value of L4 low bone density: representative example.

HealthOST uses a Convolutional Neural Network (CNN)-based AI solution that automatically identifies suspected findings suggestive of vertebral compression fractures on chest and abdominal CT scans. The AI first ensures scan eligibility by analyzing CT DICOM metadata, which includes CT modality, patient age of ≥50 years, kVp range of 80–140, and a maximum slice thickness of 3.1 mm for axial scans and 5.1 mm for sagittal scans. Once eligibility is confirmed, AI Model #1, based on a U-Net architecture, segments the spine on each axial slice, creating a structured vertebral framework. Following segmentation, AI Model #2, utilizing a RetinaNet architecture, annotates each vertebra with its corresponding label and places three height measurement lines at the anterior, middle, and posterior aspects of the vertebral body, positioned nearest to its center to facilitate fracture detection (Figure 1). In the attached figure, the AI also provided attenuation values for diagnosing osteoporosis based on low bone density, which were not assessed in our study. The percentage of vertebral height loss is determined by comparing the three different height lines for each complete vertebral body within the thoracolumbar spine. Vertebral height loss values that exceed a predefined threshold are highlighted to provide the user with clear indications of significant compression.

## 3. Results

### 3.1. Study Cohort

The dataset comprised 675 outpatient cases selected between February 2019 and March 2020, which were subsequently sent for automated image analysis using artificial intelligence (AI). Two AI algorithms were employed: one that assessed fractures above a 20% loss of vertebral body height and another one that assessed above a 25% loss of vertebral body height. A total of 65 cases were excluded from the AI analysis due to non-compliance with the algorithm requirements for five primary reasons: less than 15 cm of the spine was detected (34 cases); fewer than four vertebrae were observed (13 cases); there was an absence of a valid CT series (7 cases); there was an insufficient number of images, specifically less than 20 (8 cases); and systemic error (3 cases). Out of the remaining 610 cases, a further 20 cases were excluded during review when bone metastasis (16 cases) and spinal hardware (4 cases) were discovered, which left 590 cases for the final analysis.

Table 1 presents a demographic analysis of patients with and without vertebral fractures. Patients with fractures were older, with a mean age of 72.5 years (SD 10.7), compared to those without fractures (mean age 66.9 years, SD 9.7). Regarding gender distribution, a higher proportion of females had fractures (55.9%) compared to males (44.1%). These findings indicate a higher prevalence of fractures among older individuals and a slightly higher proportion of fractures in females relative to their total representation in the study population.

### 3.2. AI Performance at Two Thresholds

The AI software’s performance in detecting vertebral fractures was assessed using two thresholds for vertebral body height loss: a 20% cutoff and a 25% cutoff. The results are provided in three tables (Table 2, Table 3 and Table 4). The analysis was conducted for each individual vertebral body rather than per patient, allowing for the inclusion of multiple fractures occurring in single individuals. Initially, a single point was assigned for each patient without fractures, but this understated the number of vertebrae that were separately evaluated and confirmed as negative. Since the AI software excluded cases with fewer than four vertebrae, a decision was made to assign four points per negative case, ensuring a consistent representation of normal vertebral bodies in the dataset. This approach mirrors the evaluation of positive fractures, where each fractured vertebra was assessed individually, and allows for more accurate calculations of specificity and negative predictive value.

At the 20% cutoff, the AI demonstrated high sensitivity (92.0%), detecting most fractures but at the cost of low specificity (52.7%) and a high false-positive rate, leading to a low PPV (16.5%). In contrast, the 25% cutoff improved the specificity (94.2%) and PPV (51.1%), reducing false positives but lowering sensitivity (78.0%), resulting in more missed fractures. Despite these trade-offs, the NPV remained high for both thresholds (98.5% and 98.2%), indicating strong reliability in ruling out fractures.

### 3.3. False Positives

**Figure 2:**
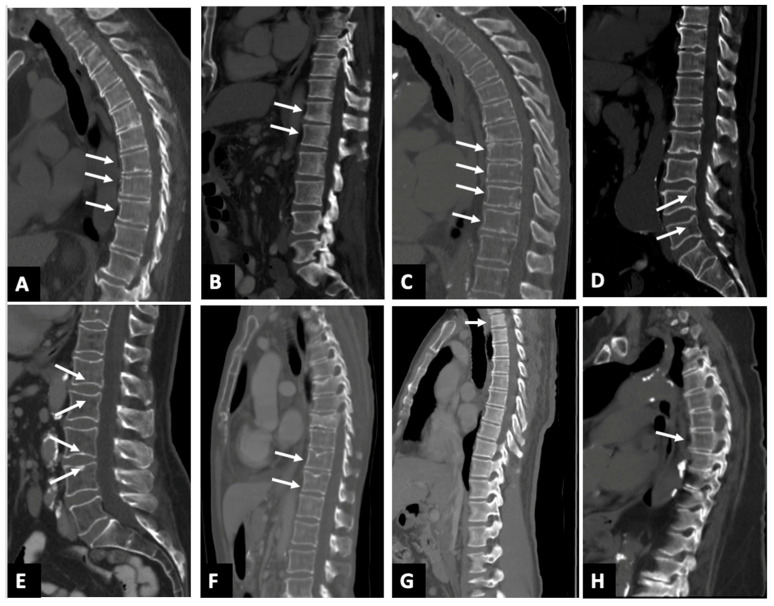
Sagittal CT images (**A**–**H**) in 8 different patients with AI software calling false positive fracture. (**A**) the white arrows show osteoarthritic wedging deformity involving vertebral bodies T8–10; (**B**) the white arrows show physiological wedging deformity involving vertebral bodies T12–L1; (**C**) the white arrows show endplate irregularities denoting Scheuermann’s disease, noted in multiple lower thoracic vertebral bodies, namely T8–11; (**D**) the white arrow shows cupids bow deformity noted in lumbar vertebral bodies L4–L5; (**E**) the white arrows show concavity/ballooned disk spaces noted in lumbar vertebral bodies L1–L4; (**F**) the white arrows show Schmorl’s node involving vertebral bodies T11–12. Fractures of T7 and T9 vertebral bodies were accurately identified; (**G**) the white arrows show edge of field of view overcalls involving the T1 vertebral body that appears normal; (**H**) the white arrows show T6 fracture overcall in a patient with scoliosis.

Large amounts of false positives (Figure 2) reported by the AI at the 20% threshold largely fall into categories such as physiological/osteoarthritic wedging, endplate irregularities, edge of field of view effects, and scoliosis. A total of 146 patients were deemed to have fractures attributed to physiological/osteoarthritic wedging, which refers to mild anterior vertebral wedging not linked to acute trauma or pathological fractures. This type of wedging can occur as part of natural spinal curvature or minor degenerative osteoarthritic changes and is often mistaken for a fracture by imaging software due to the shape of the vertebra, particularly in regions like the mid-thoracic spine and thoracolumbar junction (Figure 2A,B). False positives were also noted from endplate irregularities, such as Schmorl’s nodes, concavity/ballooned disk spaces, Cupid’s bow deformities, and Scheuermann’s disease, accounting for 43 patients (Figure 2C–F). The AI also struggled to accurately assess fractures at T1 when located at the edge of field of view effect (Figure 2G) film, leading to 36 false positives with only one confirmed fracture. Scoliosis was noted in seven patients, complicating the vertebral assessment due to altered spinal curvature that often led to either incorrect vertebral numbering/labeling or overcalling fractures (Figure 2H). Most of the AI’s false positives clustered around the 20–25% threshold. Initially, 395 out of 590 patients were flagged as potential fractures at a 20% cutoff, which reduced to 137 patients when the threshold was increased to 25%, mainly due to overdiagnosis in the aforementioned categories.

### 3.4. Detection of Missed Fractures

A secondary objective of our study was to determine the prevalence of missed vertebral compression fractures in outpatient CT scans at our institution. With a total of 150 fractures, at the 20% cutoff, radiologists identified 54.7% of fractures, leaving 68 fractures undetected. The AI software identified 60 of these previously undetected fractures, successfully detecting 88% of the fractures that radiologists had initially missed. At the 25% cutoff, radiologists detected 66.7% of fractures, leaving 50 fractures undetected. The AI software identified 46 of these previously undetected fractures, successfully detecting 92% of the fractures that radiologists had initially missed.

## 4. Discussion

This study validated the performance of the HealthOST software in detecting vertebral compression fractures on outpatient CT scans, emphasizing the impact of threshold selection on diagnostic accuracy. A 20% vertebral height loss threshold demonstrated high sensitivity (92.0%), making it an effective screening tool for minimizing missed fractures. However, its lower specificity (52.7%) results in more false positives, which can lead to overdiagnosis. This makes it ideal for health systems prioritizing early detection and maximizing fracture identification provided there is a structured workflow to manage follow-up. Conversely, the 25% threshold offers improved specificity (94.2%) and a higher positive predictive value (PPV), reducing false positives and unnecessary imaging. Institutions with limited follow-up capacity may favor the 25% threshold, while those focused on comprehensive fracture detection may opt for the 20% threshold to ensure early intervention. Importantly, the negative predictive value (NPV) remains high across both thresholds, indicating the AI’s strong ability to reliably confirm negative cases. Given the trade-off between sensitivity and specificity and the potential for false positives, particularly at lower thresholds, a secondary radiologist review is recommended to ensure diagnostic accuracy and minimize unnecessary follow-ups.

Ultimately, the selection of the optimal threshold should not only align with institutional priorities but also consider the clinical significance of mild fractures. Some studies have shown that identifying and treating mild incidental vertebral fractures reduce future fracture by facilitating earlier osteoporosis management [31,32,33]. However, there are also other studies that indicate that mild fractures alone do not significantly alter future fracture risk unless accompanied with additional osteoporosis risk factors [34,35]. While early detection at a lower threshold may allow for proactive osteoporosis management, radiologists and clinicians may choose to focus on moderate and severe fractures (>25% vertebral height loss) given their stronger predictive value for future osteoporotic fractures [36,37,38].

The AI does not assess fracture acuity, and no acute fractures were identified in this dataset, consistent with the outpatient nature of the study population. Among the 590 fractures reviewed, 580 were chronic, and 10 were classified as subacute or chronic. According to Lentle et al., morphometric criteria may be less effective than morphological criteria in fracture grading, as defined by the mABQ grading system [31]. However, due to the limitations of Nanox.AI, morphological signs were not assessed. One important point to note is that the Nanox.AI software estimates vertebral body height loss based on intravertebral measurements (comparing cortices within the same vertebrae), unlike the intervertebral measurements (comparing the affected vertebral cortex to adjacent vertebrae) often used in practice [39,40]. This discrepancy can result in the overcalling of fractures like those discussed above for physiological/osteoarthritic wedging. This discrepancy also caused variable GSQ grading between the radiologist and AI software.

The most common reason for fractures being missed by the AI was their location at the edge of the scan’s field of view, where incomplete vertebral visualization affected assessment. The second most frequent cause was borderline height loss (20–25%), which led to discrepancies between AI detection and radiologist interpretation. This is likely due to AI’s reliance on intravertebral height assessment (comparing cortices within the same vertebra), whereas radiologists typically assess fractures using an intervertebral method, comparing the affected vertebra to adjacent levels. These findings suggest that refining AI algorithms, particularly in recognizing fractures at scan boundaries and better aligning vertebral height measurement methods with radiologist practices, could enhance detection accuracy.

The initial radiologists’ report revealed significant differences in fracture detection compared to the AI. At the 20% cutoff, the radiologists detected 54.7% of fractures, leaving 68 undetected, of which the AI identified 60 (88%). At the 25% cutoff, the radiologists detected 66.7%, leaving 50 undetected, with the AI identifying 46 (92%). It is important to note that all cases were outpatient studies with unrelated clinical indications. This highlights the AI’s capability to assist in fracture detection and to supplement radiologist interpretation.

Recent studies evaluating AI applications in vertebral fracture detection have reported consistently high sensitivity and specificity, reinforcing the reliability of AI models [41,42]. For example, a deep learning system for thoracolumbar vertebral fractures on CT demonstrated a sensitivity of 95.23% and a specificity of 98.35% [43]. Systematic reviews further highlighted AI’s effectiveness with sensitivity and specificity that varied among different AI models but remained high across most studies, with sensitivity ranging from 62 to 97% and specificity ranging from 83 to 100% [44]. Additionally, another systematic review and meta-analysis evaluating machine learning models for vertebral fracture diagnosis reported a sensitivity of 93% and a specificity of 96% for osteoporotic fractures [45]. A retrospective analysis similar to our study reported that for moderate and severe (25% height loss and above) VCFs, the AI algorithm achieved 85.2% sensitivity, 92.3% specificity, a 57.8% positive predictive value, and a 98.1% negative predictive value, further demonstrating AI’s clinical utility in identifying higher-grade fractures [46]. Burns et al. also developed an automated system that achieved high sensitivity (95.7%) and a low false-positive rate for vertebral compression fracture detection, with strong Genant-based classification accuracy (accuracy 0.95; κ = 0.90) [27]. Another study evaluating a deep learning model for acute vertebral fractures on routine chest and abdominal CT scans also demonstrated high accuracy and precision, further supporting the use of AI in opportunistic screening [47]. These findings align with our results and further support AI’s role in enhancing vertebral fracture detection.

This study has some limitations. As a single-center study, its findings may not fully generalize to other populations and health care settings. Future studies should enroll larger cohorts from multiple institutions and diverse demographics to validate the performance of HealthOST across diverse patient populations. Additionally, as a retrospective study focused on outpatient CT scans, it may not capture the full spectrum of vertebral fractures, particularly those seen in acute or inpatient settings potentially affecting fracture prevalence and AI performance characteristics. A key technical limitation of the AI software is its reliance on intravertebral evaluation, where vertebral height loss is assessed within the same vertebra rather than comparing it to adjacent vertebrae (intervertebral evaluation). This can lead to discrepancies in fracture grading and overcalls, particularly in cases of physiological wedging. While scans with partial vertebral visualization may contain clinically significant findings, HealthOST requires at least four contiguous vertebrae for accurate segmentation. As a result, scans with fewer vertebrae cannot be reliably processed and are excluded, which we acknowledge as a limitation of the current software version. It is important to note that around the time of this paper’s publication, Nanox had nearly completed adjustments to its software to address edge of field of view overcalls. This highlights the ongoing evolution of Nanox.AI technology, reinforcing the notion that AI systems will continue to improve in accuracy and adaptability. Such refinements are crucial for advancing AI’s role in clinical practice, ultimately enhancing patient care and diagnostic confidence.

## 5. Conclusions

This study presents a clinical validation of the HealthOST AI software for the detection of incidental vertebral compression fractures on routine chest and abdominal CT scans. At the 20% cutoff, the AI demonstrated high sensitivity (92.0%), capturing most fractures but with lower specificity (52.7%) and a low PPV (16.5%) due to more false positives. At the 25% threshold, the specificity (94.2%) and PPV (51.1%) improved, but the sensitivity decreased (78.0%), resulting in more missed cases. These findings support the use of AI in opportunistic fracture screening, with threshold selection tailored to clinical priorities, favoring higher sensitivity for broad screening or higher specificity for confirmatory purposes. Furthermore, our secondary analysis demonstrated that the AI detected several fractures that were missed in original radiology reports, reinforcing its value as a supportive tool in routine clinical practice.

## Figures and Tables

**Table 1:** Patient demographics.

Number of Patients	Fractures	Normal	Total
Mean age, years (SD) [CI]	72.5 (10.7) [70.3–74.7]	66.9 (9.7) [66.0–67.8]	67.8 (10.1) [67.0–68.6]
Number of males (%) [CI]	41 (44.1%) [0.34–0.54]	273 (54.9%) [0.51–0.59]	314 (53.2%) [0.49–0.57]
Number of females (%) [CI]	52 (55.9%) [0.46–0.66]	224 (45.1%) [0.41–0.50]	276 (46.8%) [0.43–0.51]
Total Patients (%) [CI]	93 (15.8%) [0.13–0.19]	497 (84.2%) [0.81–0.87]	590 (100%) [0.99–1.0]

SD—Standard Deviation; CI—Confidence Interval.

**Table 2:** Results for 20% cutoff AI fracture detection.

AI	Fracture Present		Fracture Absent		Total
Positive	True positive	138	False Positive	699	837
Negative	False Negative	12	True Negative	780	792
Total		150		1479	

**Table 3:** Results for 25% cutoff AI fracture detection.

AI	Fracture Present		Fracture Absent		Total
Positive	True positive	117	False Positive	112	229
Negative	False Negative	33	True Negative	1812	1845
Total		150		1924	

**Table 4:** Calculated metrics.

Metrics	20% AI Cutoff [CI]	25% AI Cutoff [CI]
Sensitivity	92.0% [0.87–0.95]	78.0% [0.71–0.84]
Specificity	52.7% [0.50–0.55]	94.2% [0.93–0.95]
Positive Predictive Value (PPV)	16.5% [0.14–0.19]	51.1% [0.45–0.58]
Negative Predictive Value (NPV)	98.5% [0.97–0.99]	98.2% [0.98–0.99]

CI—Confidence Interval.

## Data Availability

Restrictions apply to the datasets presented in this article. The datasets are not readily available because they are governed by institutional and ethical restrictions under our research ethics board (REB) and because portions of the data were generated using proprietary AI software from Nanox.AI. Requests to access the datasets should be directed to the corresponding author.

## Abbreviations

The following abbreviations are used in this manuscript:

VCF	Vertebral compression fractures
PPV	Positive predictive value
NPV	Negative predictive value
AI	Artificial intelligence
GSQ	Genant semiquantitative
QM	Quantitative morphometry
mABQ	Morphological algorithm-based qualitative
